# Expression of GADS enhances FLT3-induced mitogenic signaling

**DOI:** 10.18632/oncotarget.7415

**Published:** 2016-02-15

**Authors:** Rohit A. Chougule, Eugenia Cordero, Sausan A. Moharram, Kristian Pietras, Lars Rönnstrand, Julhash U. Kazi

**Affiliations:** ^1^ Division of Translational Cancer Research, Department of Laboratory Medicine, Lund University, Lund, Sweden; ^2^ Lund Stem Cell Center, Department of Laboratory Medicine, Lund University, Lund, Sweden; ^3^ Lund University Cancer Center, Medicon Village, Lund, Sweden

**Keywords:** GRAP2, AML, STAT5, FLT3-ITD, RTK

## Abstract

GADS is a member of a family of SH2 and SH3 domain-containing adaptors that functions in tyrosine kinase-mediated signaling cascades. Its expression is largely restricted to hematopoietic tissues and cell lines. Therefore, GADS is mainly involved in leukocyte-specific protein tyrosine kinase signaling. GADS is known to interact with tyrosine-phosphorylated SHC, BCR-ABL and KIT. The SH2 domain of GADS has a similar binding specificity to that of GRB2 but its SH3 domain displays a different binding specificity, and thus it is involved in other downstream signaling pathways than GRB2. In the present study, we examined the role of GADS in FLT3 signaling. FLT3 is a type III receptor tyrosine kinase, which is mutated in more than 30% of acute myeloid leukemia (AML) and the most common mutations is the internal tandem duplication (ITD) mutations. We observed that expression of GADS enhanced oncogenic FLT3-ITD-induced cell proliferation and colony formation *in vitro*. In a mouse xenograft model, GADS accelerated FLT3-ITD-dependent tumor formation. Furthermore, expression of GADS induced a transcriptional program leading to upregulation of MYC and mTORC1 target genes. GADS localizes to the cell membrane and strongly binds to ligand-stimulated wild-type FLT3 or is constitutively associated with the oncogenic mutant FLT3-ITD. We mapped the binding sites in FLT3 to pY955 and pY969 which overlaps with the GRB2 binding sites. Expression of GADS enhanced FLT3-mediated phosphorylation of AKT, ERK1/2, p38 and STAT5. Taken together, our data suggests that GADS is an important downstream component of FLT3 signaling and expression of GADS potentiates FLT3-mediated mitogenic signaling.

## INTRODUCTION

The receptor tyrosine kinase FLT3 belongs to the type III receptor tyrosine kinase family and is preferentially expressed in acute myeloid leukemia (AML) and acute lymphocytic leukemia (ALL) cells in addition to hematopoietic stem cells, brain, placenta and liver [1, 2]. FLT3 is known to play an important role in the survival, proliferation and differentiation of normal hematopoietic cells and progenitor cells as well as leukemic cells [2–6]. Knockout mice lacking the *FLT3* gene have a normal hematopoietic cell population. However, they exhibit a reduced number of B lymphoid progenitors and multipotent stem cells [7]. AML patients very often carry a mutation in the *FLT3* gene and the FLT3-ITD (internal tandem duplication) mutations occurring in the juxtamembrane region of the receptor are the most common. These mutations, and other oncogenic mutations in the kinase domain of FLT3, have been reported in approximately 35% of AML patients. While wild-type FLT3 is dependent on its ligand, FL, for activation, oncogenic FLT3 mutants are constitutively active and not dependent on ligand for their activation. Activation of FLT3 in turn activates several signaling proteins, including PI3-kinase, the MAP-kinases ERK1/2 and p38, and STAT5 [8–10]. Binding of its ligand to the extracellular domain of FLT3 induces receptor dimerization, activation and autophosphorylation of several cytoplasmic tyrosine residues, which provide docking sites for a number of signal transducing proteins containing SH2 domains [11, 12]. A majority of hematopoietic receptor tyrosine kinases are dependent on adaptor proteins for the activation of downstream signaling pathways. Several adaptor proteins including GRB2, GADS, SHC and NCK have been found to directly bind to the activated receptors through their SH2-domain [13–15]. These adaptor proteins function to recruit other cytosolic signaling molecules to the activated receptors via their other domains and, there by, initiate tyrosine kinase-dependent signaling events [11]. We and other investigators have identified several FLT3-associating proteins that are involved in regulating signaling downstream of FLT3. While many of the interacting proteins, including SLAP [16, 17], GRB10 [18, 19], GAB2 [20], GRB2 [20], SHP2 [21], SYK [22], and SRC, act as enhancers of FLT3 signaling, others such as SOCS2 [23, 24], SOCS6 [25, 26], CSK [27] and LNK [28] negatively regulate downstream signaling. Apart from these interacting proteins, other cytosolic proteins also regulate FLT3 signaling. Recently we demonstrated that BEX1, a brain X-linked family protein negatively regulates FLT3 signaling by modulating FLT3-induced AKT activation [29].

Receptor tyrosine kinase signaling is tightly regulated by a variety of intermediate adaptor proteins, but in most cases, their site of interaction and roles in the physiological events are not clear. GRB2-related adaptor protein 2 (GRAP2), also known as GRB2-related adaptor downstream of SHC (GADS), is among one of them and is encoded by the *GRAP2* gene. GADS is a member of the family of SH2 and SH3 domain-containing adaptor proteins whose expression is mainly restricted to hematopoietic tissues, including bone marrow, lymph node, and spleen [30–32]. GADS plays an important role in mitogenic signaling from RET leading to activation of the transcription factor NF-κB [33]. In addition, GADS is known to play a major role in T cell development [34] and T cell receptor (TCR) signaling [35, 36]. Emerging evidence suggests that GADS may also play additional roles in antigen-receptor signaling and receptor tyrosine kinase-mediated signaling in other hematopoietic lineages. GADS has also been reported to be associated with other proteins including BCR-ABL, CD28, SHP2 and KIT [30, 37, 38]. However, the physiological role of these interactions remains mostly unknown. In this study, we show that GADS interacts with FLT3 and enhances FLT3 downstream signaling, resulting in aberrant cell proliferation, colony and tumor formation.

## RESULTS

### GADS expression potentiates FLT3-ITD-induced cell proliferation and colony formation

**Figure 1:**
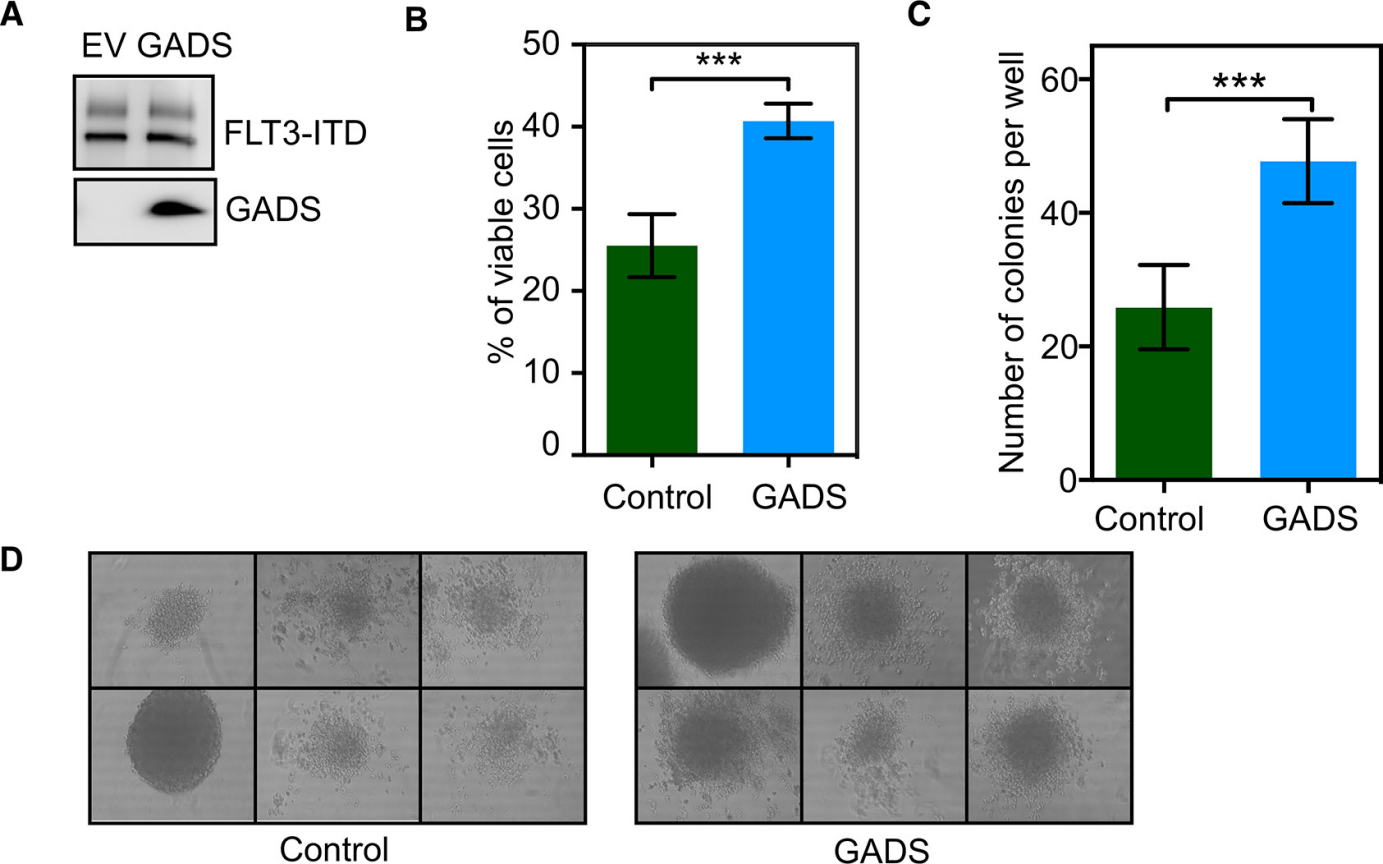
GADS expression significantly contributed to cell proliferation and colony formation Ba/F3/FLT3-ITD cells stably transfected with GADS or empty vector were washed three times with RPMI-1640 to remove IL3. (**A**) An equal amount of cells were lysed and the lysate was used to check FLT3 and GADS expression. (**B**) FLT3-ITD-dependent cell proliferation in the presence or absence of GADS expression was measured after 48 hours using PrestoBlue cell viability assay. (**C**–**D**) Around 500 cells were seeded in semi-solid medium followed by counting colonies after seven days.

To understand the role of GADS in oncogenic FLT3-ITD signaling, we generated Ba/F3 cells expressing FLT3-ITD and GADS or empty control vector (Figure 1A). The mouse proB cell Ba/F3 lacks expression of FLT3 and GADS, and is therefore a useful model system for this study. Initially, we checked whether GADS plays a role in FLT3-ITD-mediated cell proliferation. We observed that cells expressing GADS have enhanced FLT3-ITD-induced cell proliferation compared to empty vector-transfected cells (Figure 1B). However, GADS expression was unable to reduce the level of apoptosis seen upon cytokine depletion (data not shown) suggesting that GADS plays a role in FLT3-ITD-induced cell proliferation but does not contribute to cell survival. In addition, we observed that GADS significantly enhanced FLT3-ITD-dependent colony formation in semi-solid medium (Figure 1C and 1D).

### GADS expression leads to enhanced tumor formation in a mouse xenograft model

**Figure 2:**
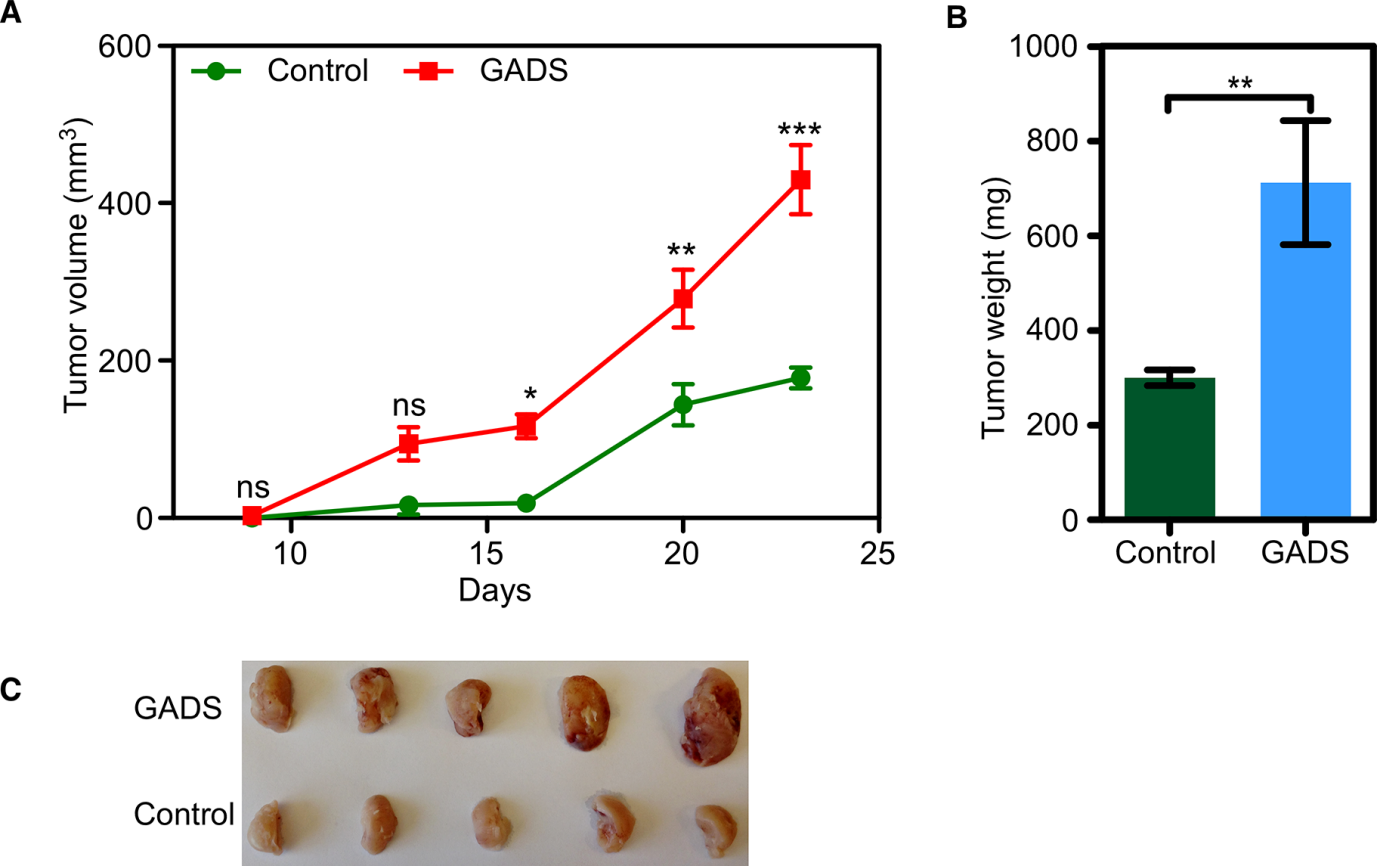
GADS enhances tumor growth in a mouse xenograft model Cells were washed three times with cold PBS to remove IL3. (**A**–**C**). Cells expressing GADS or empty vector were xenografted into mice and tumor growth was monitored for 23 days. Tumor volume (A) was measured twice a week and tumor weight (B) was measured after sacrificing the animals.

Because GADS expression potentiated cell proliferation and colony formation, we aimed to check whether GADS expression enhances FLT3-ITD-induced tumor formation in mice. We xenografted immunodeficient mice with 200,000 cells. We observed that GADS expression significantly enhanced tumor volume seen at 16 days after the establishment of xenografts (Figure 2A). Tumor weight was also significantly increased in mice xenografted with GADS expressing cell (Figure 2B and 2C).

### GADS expression correlates with enrichment of MYC and mTORC1 targets

**Figure 3:**
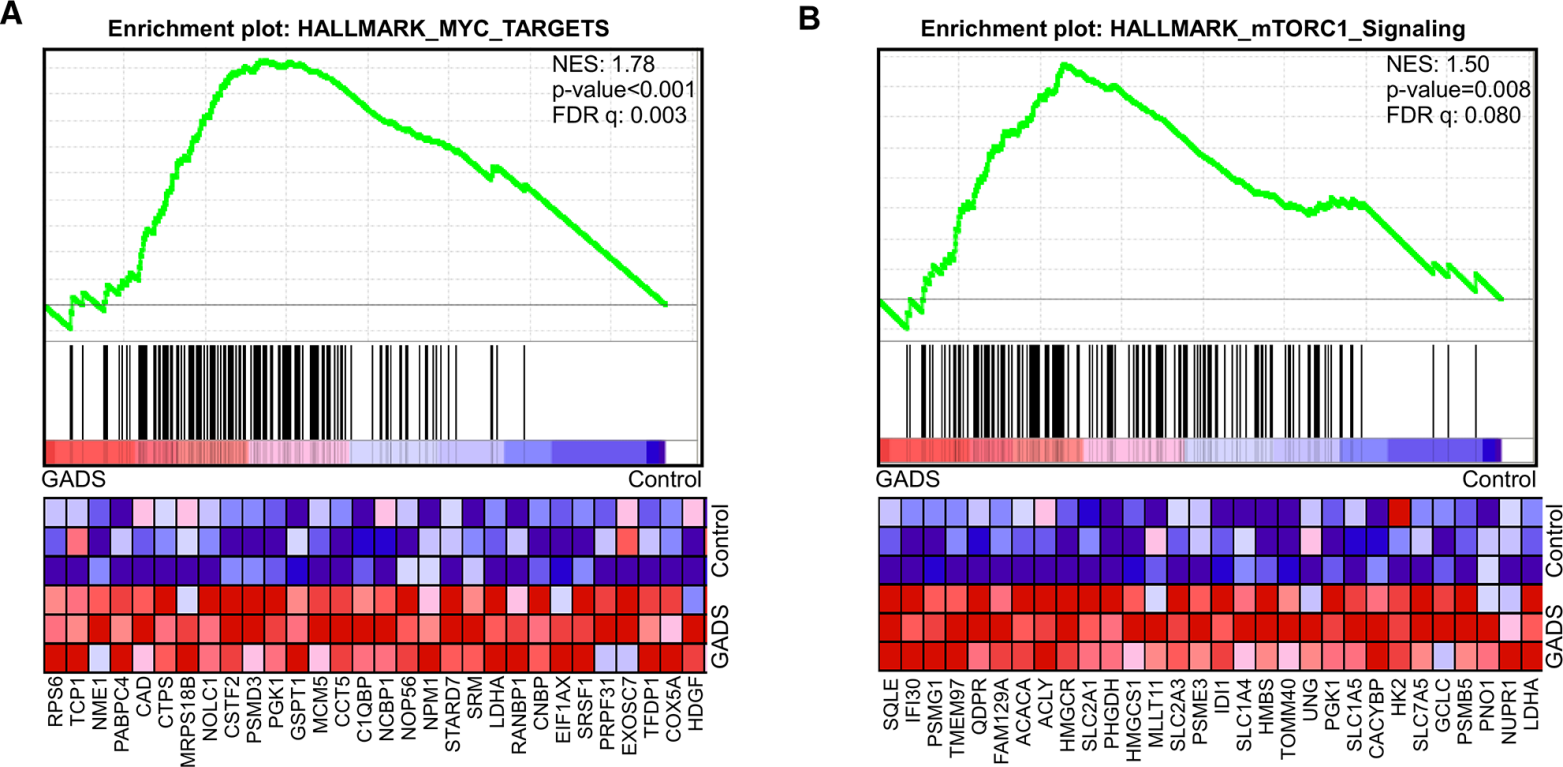
Gene set enrichment analysis (GSEA) showed enrichment of oncogenic pathways in GADS expressing cells Gene expression data from microarray analysis using GADS expressing cells display enrichment in MYC target genes (**A**) and MTORC1 target genes (**B**).

To understand how GADS regulates the FLT3-ITD-induced transcriptional program, we analyzed mRNA expression of Ba/F3-FLT3-ITD/empty vector and Ba/F3-FLT3-ITD/GADS cells. We extracted total RNA and analyzed by Affymetrix GeneChip Mouse Gene 2.0 ST arrays. We observed that GADS expression led to upregulation of a subgroup of genes regulated by MYC (Figure 3A) as well as genes up-regulated through activation of mTORC1 (Figure 3B).

### GADS interacts with the FLT3 receptor in response to FL stimulation

**Figure 4:**
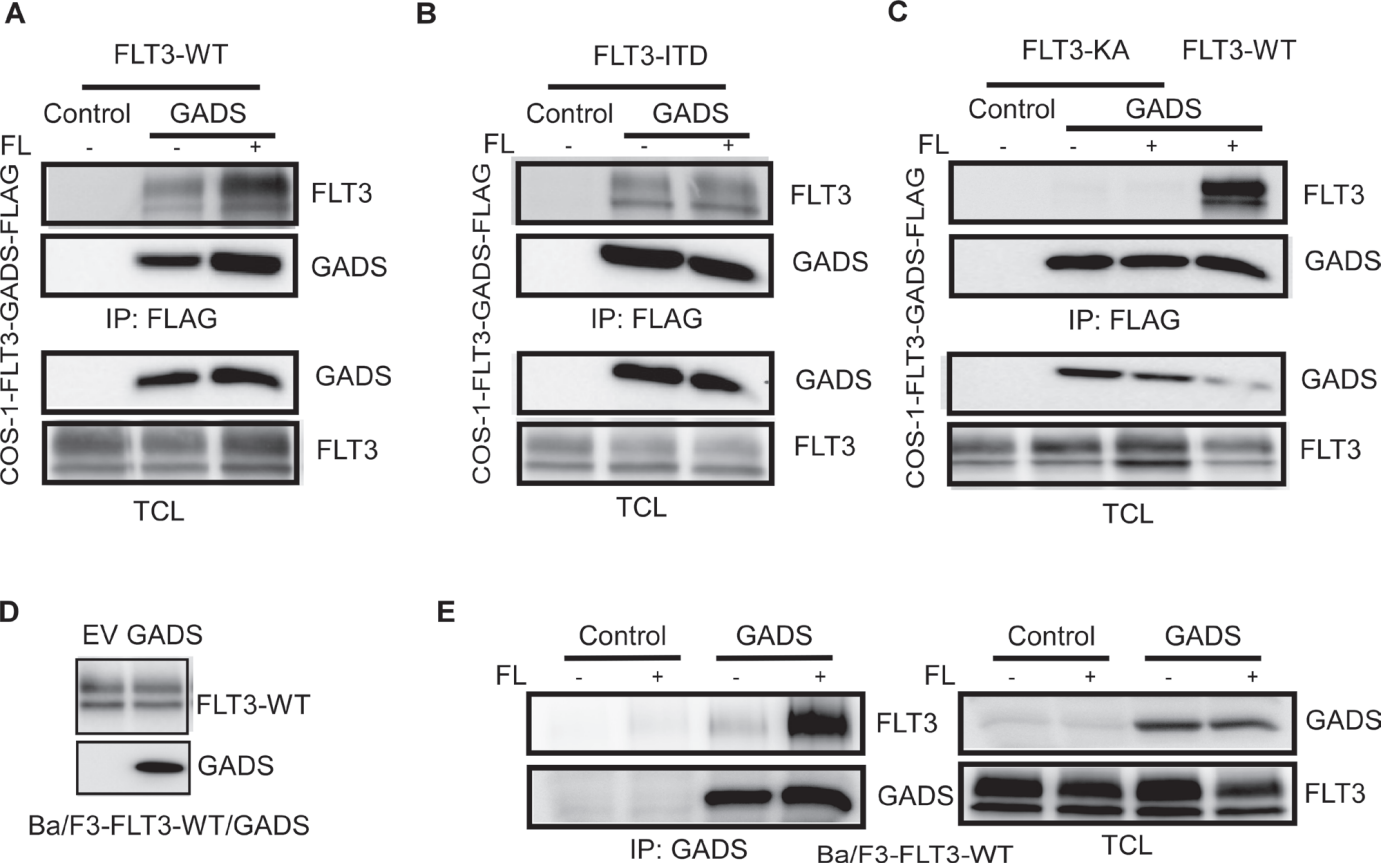
GADS binds with FLT3 in response to ligand stimulation COS1 cells were transfected with plasmids expressing FLAG-tagged GADS or empty vector and FLT3-WT (**A**) or FLT3-ITD (**B**) or FLT3-K644A/FLT3-WT (**C**). FLT3-K644A is a kinase-dead mutant of FLT3. Cells were stimulated or not with 100 ng/ml FL for five minutes before lysis. Lysates were subjected to anti-FLAG immunoprecipitation followed by Western blotting analysis (**D**) Expression of FLT3-WT and GADS were verified by Western blotting using anti-FLT3 and anti-FLAG antibodies. (**E**) Ba/F3 cells expressing FLT3-WT and empty vector or GADS were stimulated for 5 minutes. Cells were lysed and were subjected to immunoprecipitation with an anti-GADS antibody.

GADS associates with the tyrosine kinases BCR-ABL and KIT [30]. FLT3 belongs to the same family of receptor tyrosine kinases as KIT and, therefore, we investigated whether GADS also associates with FLT3. We co-transfected FLAG-tagged GADS together with wild-type FLT3 in COS-1 cells. Cells were subjected to FL stimulation before lysis. We observed a strong association of GADS with FLT3 in response to ligand stimulation (Figure 4A). While wild-type FLT3 displayed a dependence of ligand stimulation, oncogenic FLT3-ITD showed constitutive association with GADS (Figure 4B). Furthermore, a kinase-dead FLT3 mutant (FLT3-K644A) was unable to associate with FLT3 (Figure 4C) suggesting that FLT3 activation is required for association of GADS with FLT3. Furthermore, we generated Ba/F3-FLT3-WT/empty vector or Ba/F3-FLT3-WT/GADS cell lines (Figure 4D), and using an anti-GADS antibody we further observed that the GADS association to wild type FLT3 is highly dependent on FL stimulation (Figure 4E).

### GADS associates with FLT3 through its SH2 domain

**Figure 5:**
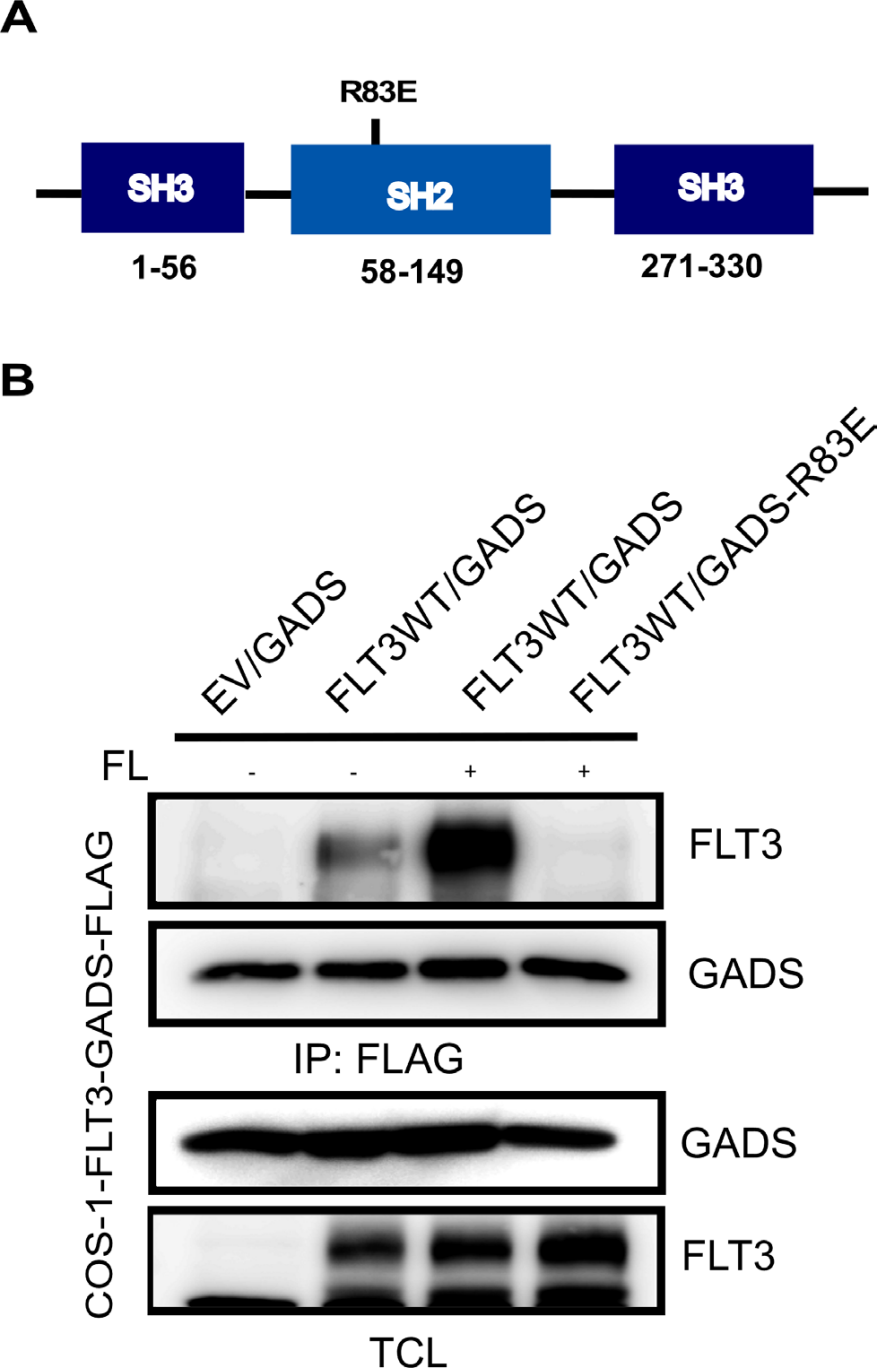
The GADS-SH2 domain associated with FLT3 (**A**) Schematic representation of GADS domains. (**B**) COS1 cells were transfected with plasmids expressing wild-type FLT3 and FLAG-tagged GADS or GADS-R83E. Cells were stimulated or not with 100 ng/ml FL for five minutes before lysis. Lysates were subjected to anti-FLAG immunoprecipitation followed by Western blotting analysis.

As we observed that FL-stimulation is required for association of wild-type FLT3 and GADS and that the kinase activity of FLT3 is required for this binding, we hypothesized that FLT3 associates with GADS through its phosphotyrosine residues and the SH2 domain of GADS. In GADS, there is an SH2 domain located between the two SH3 domains (Figure 5A). The SH2 domain creates a positively charged pocket where the negatively charged phosphotyrosine residue binds. An arginine residue is critical for the positively charged pocket and in the case of GADS, Arg^83^ is known to be the critical residue. To test our hypothesis we generated a GADS-R83E mutant. We observed that mutation of Arg^83^ completely abolished FLT3 and GADS interaction (Figure 5B) suggesting that GADS associated with FLT3 through its SH2 domain.

### The GADS binding sites in FLT3 overlap with the GRB2 binding sites

**Figure 6:**
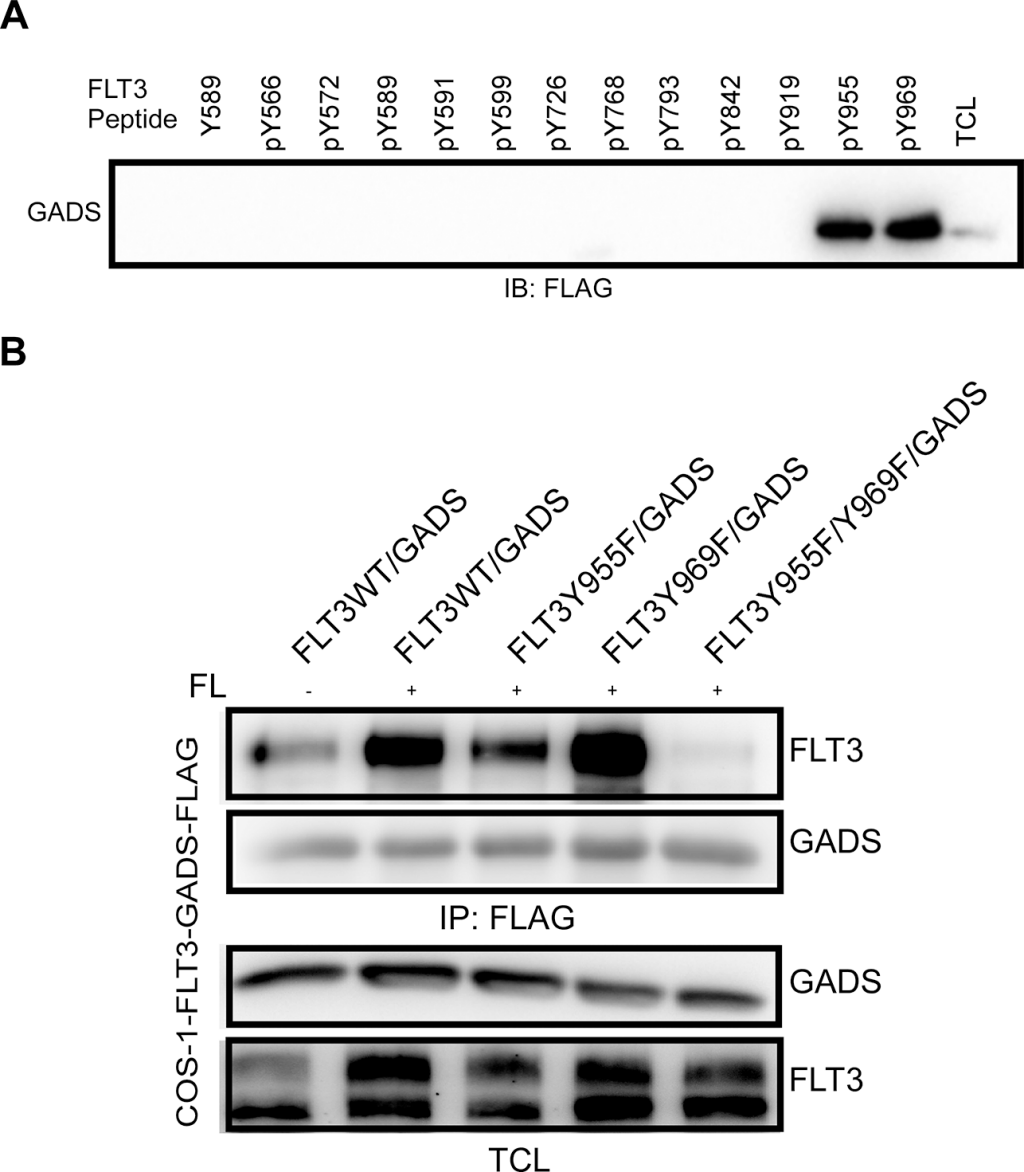
GADS associates with FLT3 through Y955 and Y969 residues (**A**) Phosphopeptides corresponding to the known or predicted tyrosine phosphorylation sites in FLT3 were used to pull down GADS from cell lysates. (**B**) COS1 cells were transfected with plasmids expressing FLAG-tagged GADS or FLT3 mutants. Cells were stimulated or not with 100 ng/ml FL for five minutes before lysis. Lysates were subjected to anti-FLAG immunoprecipitation followed by Western blotting analysis.

GADS is known to associate with other proteins containing a pY-X-N-X motif [37–39]. FLT3 has several such phosphotyrosine motifs. To determine the specific residues in FLT3 that interact with GADS, we used synthetic tyrosine phosphorylated peptides corresponding to known or predicted tyrosine phosphorylation sites in FLT3. The individual peptides were immobilized to UltraLink beads and used to pull-down FLAG-tagged GADS from cell lysates. Western blotting with an anti-FLAG antibody identified pY955 and pY969 residues as potential GADS binding sites in FLT3 (Figure 6A). These sites are also known to be binding sites for GRB2 [20, 40]. To verify the binding sites of GADS in FLT3, we generated FLT3-Y955F, FLT3-Y969F and FLT3-Y955F-Y969F mutants. While mutation at Y955F had a major impact on FLT3-GADS interaction, FLT3-Y969F did not display any difference in interaction compared to wild-type FLT3 (Figure 6B). However, mutations of both sites completely abolished the association suggesting that GADS has higher affinity to the FLT3-Y955 site and Y969 acts as a secondary binding site.

### GADS localizes to the cell surface with FLT3 but does not affect FLT3 stability in Ba/F3 cells

**Figure 7:**
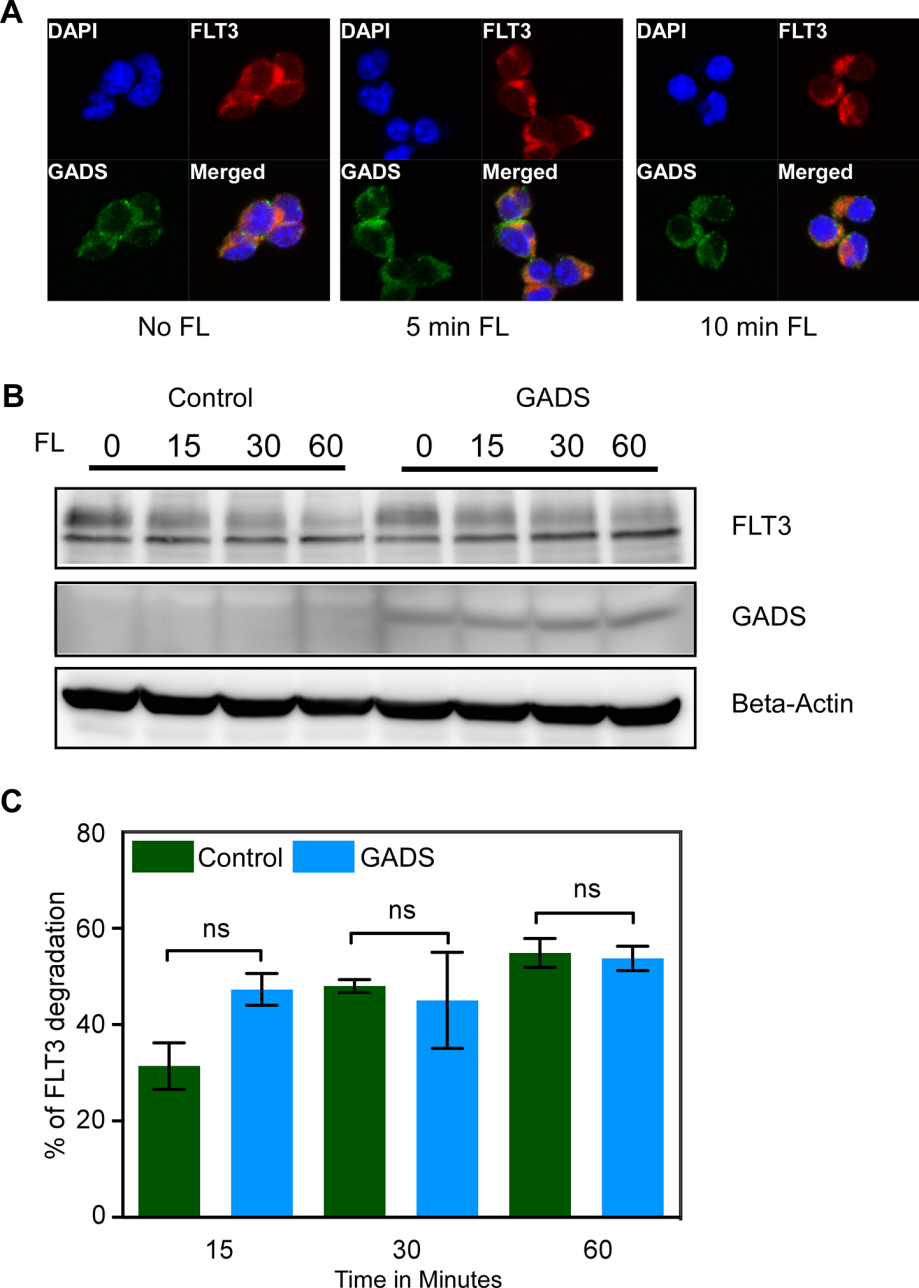
GADS localized with FLT3 to the cell surface but did not alter FLT3 stability (**A**) Ba/F3-FLT3/Empty vector and Ba/F3-FLT3/GADS cells were stimulated with 100 ng/ml FL for different time points before lysis. Cells were fixed, permeabilized, blocked and stained using PE-conjugated anti-FLT3 and Alexa Fluor 647-conjugated anti-FLAG antibody and then analyzed by confocal microscopy. (**B**) Ba/F3-FLT3/Empty vector and Ba/F3-FLT3/GADS cells were stimulated with 100 ng/ml FL for different time points before lysis. Lysates were subjected to Western blotting analysis. (**C**) Multiple blots were quantified using ImageJ and values were analyzed using GraphPad. Ns, not significant.

As GADS associates with FLT3, we hypothesized that GADS might localize to the cell surface. To address this question, we checked localization of GADS and FLT3 using confocal microscopy. We observed that GADS localized to the cell surface together with FLT3 and that FL stimulation increased the colocalization between GADS and FLT3 (Figure 7A). Many receptor-interacting proteins alter receptor stability by either recruiting ubiquitin ligases or by competing with ubiquitin ligases for the association to the receptor. We have previously shown that GRB2 is involved in degradation of KIT by acting as an adapter recruiting the ubiquitin E3 ligase CBL to the receptor [41]. Since FLT3 displays similar structure to KIT and since GRB2 shares binding sites with GADS in FLT3, we asked whether GADS plays a role in regulating FLT3 stability. However, we have not seen any significant alteration of FLT3 ubiquitination or tyrosine phosphorylation in GADS expressing cells compared to the empty vector (data not shown). Furthermore, FL stimulation in the presence of cycloheximide, an inhibitor of protein synthesis, did not result in a significant difference in FLT3 degradation (Figure 7B and 7C). Therefore, we suggest that GADS association with FLT3 has no role in regulating FLT3 stability.

### GADS expression potentiates FLT3 downstream signaling

**Figure 8:**
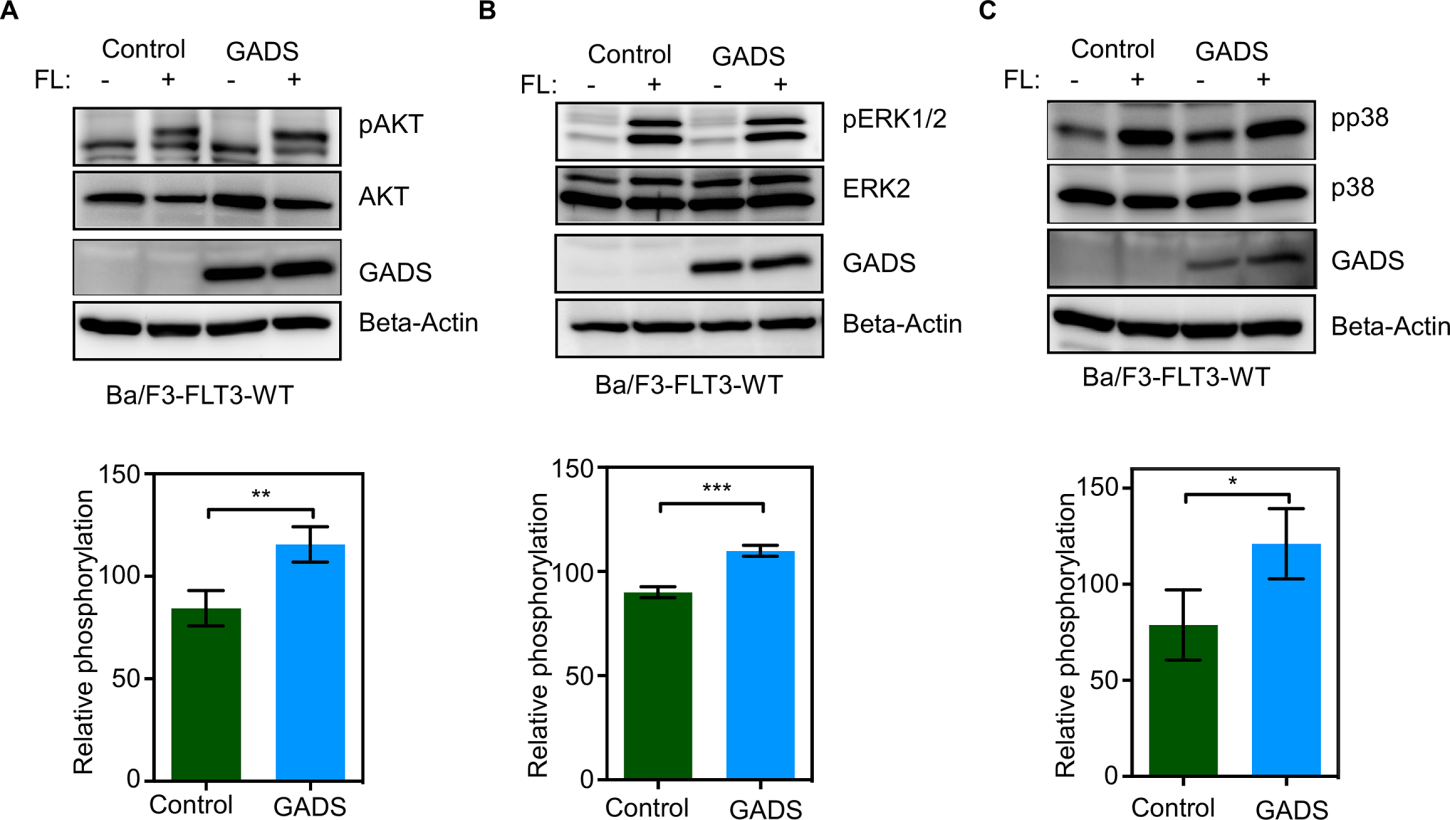
GADS expression enhances FLT3-induced signaling (**A**–**C**) Ba/F3-FLT3/Empty vector and Ba/F3-FLT3/GADS cells were serum- and cytokine-starved for four hours and stimulated with 100 ng/ml FL for five minutes before lysis. Lysates were subjected to Western blotting analysis. Multiple blots were quantified using ImageJ and values were analyzed using GraphPad and one representive blot is shown. **P < 0.01, ***P < 0.001.

Binding of FL induces dimerization of FLT3, activation of its intrinsic tyrosine kinase and activation of several downstream signaling pathways including the PI3K-AKT pathway, the RAS/ERK pathway and the p38 pathway [1]. To study the effect of GADS on these pathways, we used Ba/F3-FLT3-WT/empty vector and Ba/F3-FLT3-WT/GADS cell lines. We examined phosphorylation of AKT, ERK 1/2 and p38 by Western blotting using phospho-specific antibodies. Expression of GADS in Ba/F3-FLT3-WT cells led to a moderate increase in ligand-stimulated AKT phosphorylation (Figure 8A), ERK 1/2 phosphorylation (Figure 8B) as well as p38 phosphorylation (Figure 8C).

### GADS expression enhances FLT3-ITD-mediated STAT5 phosphorylation

**Figure 9:**
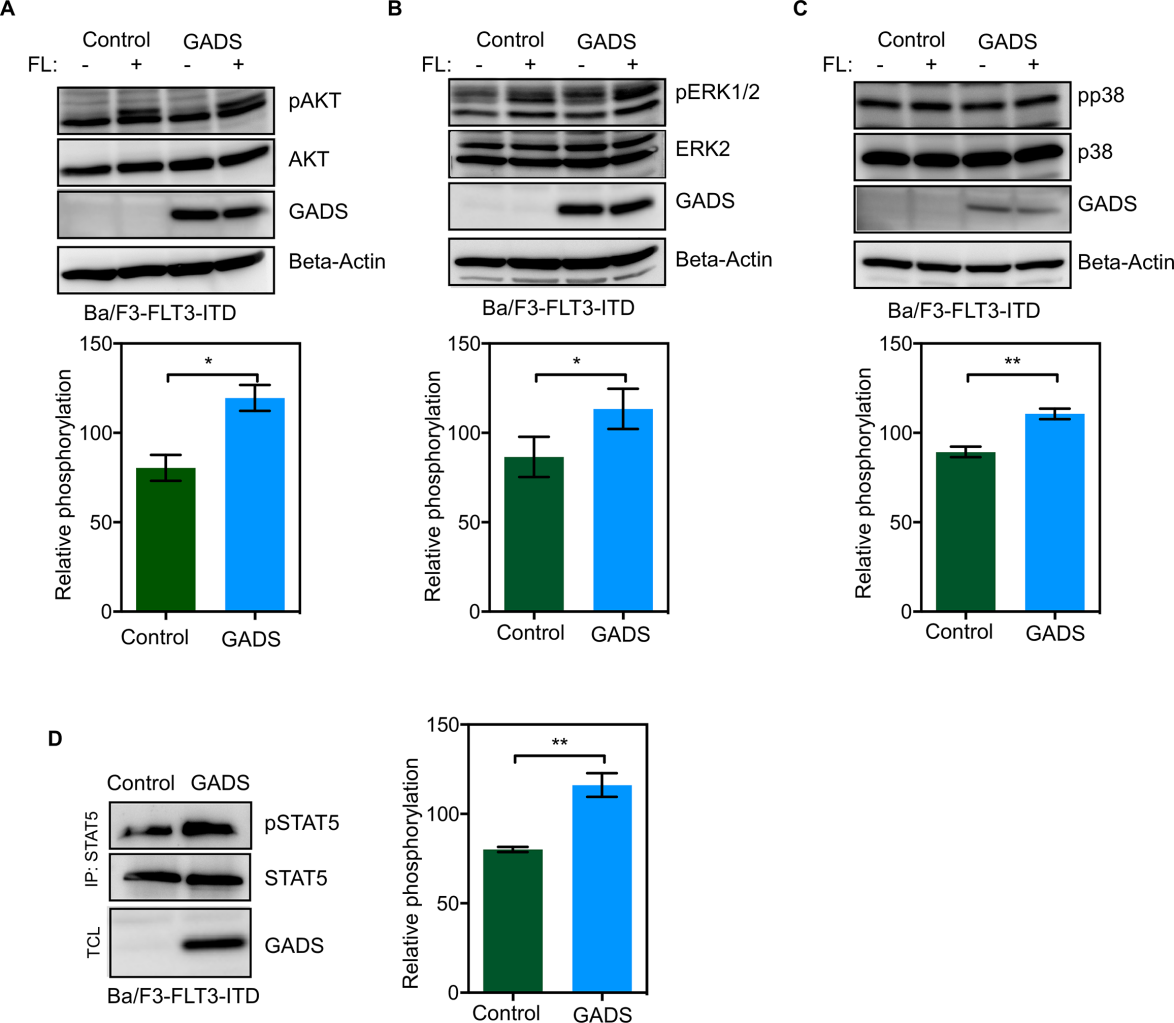
GADS expression enhances FLT3-ITD-induced STAT5 signaling (**A**–**D**) Ba/F3-FLT3/Empty vector and Ba/F3-FLT3/GADS cells were serum- and cytokine-starved for four hours and stimulated with 100 ng/ml FL for five minutes before lysis. Lysates were subjected to Western blotting analysis. Multiple blots were quantified using ImageJ and values were analyzed using GraphPad. *P < 0.05, **P < 0.01.

To understand the impact of GADS expression on oncogenic FLT3, we used Ba/F3-FLT3-ITD/empty vector and BaF3-FLT3-ITD/GADS cells. FLT3-ITD mutations lead to constitutive activation of the FLT3 kinase. Mutations in the FLT3 juxtamembrane domain have, based on the crystal structure of the FLT3 kinase domain, been predicted to result in loss of the autoinhibitory binding of the juxtamembrane domain to the kinase domain. This subsequently results in constitutive activation of its intrinsic tyrosine kinase activity and activation downstream proliferative signaling pathways, including the RAS/MAPK kinase (MEK)/extracellular signal-regulated kinase (ERK) pathway and PI3K/AKT pathway [42]. In addition, and in contrast to wild-type FLT3 signaling, FLT3-ITD potently activates the STAT5 pathway [43, 44]. In the present study we observed a moderate increase in phosphorylation of AKT (Figure 9A), ERK 1/2 (Figure 9B) and p38 (Figure 9C) in Ba/F3-FLT3-ITD/GADS cells. Furthermore, Ba/F3-FLT3-ITD cells expressing GADS displayed significantly higher STAT5 phosphorylation compared to cells transfected with empty vector (Figure 9D).

## DISCUSSION

Signaling cascades downstream of FLT3 involves the activation of multiple cytoplasmic signaling molecules by several different mechanisms, including tyrosine phosphorylation. Adaptor proteins with SH2/SH3 domains have been proposed to play an important role in signaling and in malignant transformation by deregulated tyrosine kinases. GADS is an adaptor protein known to bind several receptor tyrosine kinases via its SH2 domain and the binding motif YXN [37–39], and bridges between the receptor and cytosolic proteins in order to pass signals to the downstream effectors. In this report, we observed that GADS associated with FLT3 through phosphotyrosine residues 955 and 969, which are identical to the GRB2 binding sites in FLT3, and enhances FLT3-induced AKT, ERK, p38 and STAT5 phosphorylation [20].

Out of the three GRB2 family adaptor proteins, GRB2 is ubiquitously expressed. Expression of GRAP and GADS is restricted to hematopoietic cells including myeloid lineages [45]. Since almost all AML clones express FLT3 and a subgroup of AML patients carries mutations in FLT3, it is likely that GADS play a role in AML by regulating FLT3 signaling. Although it has been suggested that the SH2 domains of GRB2 and GADS have similar affinity to the phosphotyrosine motifs pYXNX, we observed that GADS is selective to the motif pYQNX. While GRB2 binds to pY768 (pYENQ), pY955 (pYQNV) and pY969 (pYQNR) [2], in contrast, GADS binds only to pY955 (pYQNV) and pY969 (pYQNR). Furthermore, the binding specificity of the GADS SH3 domain appears to be distinct from the SH3 domain of GRB2 [30] suggesting that GADS play a role that differs from that of GRB2. While the GRB2 SH3 domain is able to associate with SOS proteins and thereby activate the RAS/ERK pathway, the GADS SH3 domain does not bind SOS [30]. Similarly GRB2 was able to recruit CBL leading to increased ubiquitination and degradation of KIT [41], but we did not see any difference in FLT3 stability or ubiquitination in GADS expressing cells, further suggesting that the GADS SH3 domain displays differential affinity to the associating proteins, compared to GRB2, which is in agreement with what other investigators have found [30].

Like GRB2, expression of GADS potentiates FLT3 downstream signaling resulting in elevated phosphorylation of AKT, ERK, p38 and STAT5. Given the fact that, in contrast to GRB2, GADS has been reported not to associate with CBL [30], an involvement of ubiquitination in these responses is unlikely. Taken together, our study suggests that GADS is an important component of FLT3 signaling in AML and inhibition of GADS association with FLT3 can be a potential drug target in AML.

## MATERIALS AND METHODS

### Plasmids

All FLT3 plasmids were previously described [25]. A modified pcDNA3.1 vector (pcFLAG) [27] was used to subclone the open reading frame of the human *GRAP2* gene (BC025692) using BamHI and XhoI cloning sites. The GADS-R83E mutant was generated by site-directed mutagenesis using the QuikChange mutagenesis XL kit (Stratagene, La Jolla, CA). pMSCVneo vector was used for retroviral transduction.

### Antibodies

Anti-phospho-AKT, anti-phospho-ERK, Anti-phospho p38, anti-AKT, anti-ERK and anti-p38 antibodies were described earlier [26, 46]. Mouse anti-FLAG and anti-β-actin antibodies were purchased from Sigma. Phycoerythrin (PE)-conjugated anti-FLT3 was from BD Biosciences. Rabbit polyclonal anti-phospho-ERK1/2 and goat polyclonal anti-AKT antibodies were from Santa Cruz Biotechnology. The general phosphotyrosine antibody 4G10 was from Millipore. Rabbit polyclonal anti-AKT antibody was from Epitomics. Mouse monoclonal anti-phospho-p38 and anti-p38 antibodies were from BD Transduction Laboratories.

### Chemicals

The transfection reagents Lipofectamine 2000 was from Invitrogen. Cycloheximide was from Sigma. FLT3 ligand (FL) was purchased from ProSpec-Tany.

### Cell culture

The COS-1 cells were cultured in Dulbecco's modified Eagle's medium (DMEM) supplemented with 10% fetal bovine serum (FBS) and 1% penicillin. Ba/F3 cells were cultured in RPMI 1640 medium supplemented with 10% heat-inactivated FBS and 10 ng/ml recombinant murine interleukin-3 (IL-3) as recommended previously [47].

### Transient transfection

The Lipofectamine 2000 transfection reagent was used for transient transfections. Cells were seeded in the evening for next morning transfections. For co-transfections, 5 μg of total plasmids (2 μg receptor plasmid, 3 μg GADS plasmid) and 10 μl of Lipofectamine 2000 were used. Transfection was carried out in a 10 cm Petri-dish with 5 ml of complete medium. Six hours after transfection, the cells were washed with PBS for three times and cultured in serum-free medium for 18 h before stimulation and/or lysis.

### Retroviral transduction

The retroviral transduction procedure has previously been described [19]. In brief, packaging EcoPack cells were transfected with the respective plasmids using Lipofectamine 2000 as per the manufacturers' protocol. Six hours after transfection, the medium was replaced with fresh complete growth medium. The cells were then grown for 48 h to produce viral particles. Ba/F3 cells were cultured in medium containing viral particles for 24 h and were grown for an additional 48 h in complete Ba/F3 medium before starting puromycin selection.

### Immunoprecipitation, SDS-PAGE and western blotting

Ba/F3 cells were kept in IL-3 and serum-free medium for 4 h before stimulation. After stimulation, cells were washed with ice-cold PBS. Cells were then lysed in 1% Triton X100 buffer on ice. Lysates were processed for immunoprecipitation, SDS-PAGE and Western blotting [48].

### FLT3 degradation

Cells were serum-starved for 4 h and then incubated with cycloheximide for 30 min. Cells were then stimulated with FL for the indicated period of time in the presence of cycloheximide and processed for lysis. Cell lysates were used for analysis.

### Cell proliferation

Ba/F3-FLT3-ITD/empty vector and Ba/F3-FLT3-ITD/GADS cells were washed three times with PBS and seeded in 96-well plates (10,000 cells/well). Cells were incubated with or without 100 ng/ml FL for 46 h. Then 10 μl of PrestoBlue (Molecular Probes) was added to each well, followed by 2 h of incubation. Absorbance at 570 nm and 600 nm was measured using a 96-well plate reader and cell viability was calculated according to the manufacturer's protocol.

### Colony formation assay

Ba/F3 cells were washed three times with RPMI 1640. Cells were mixed with methylcellulose medium and seeded in a 24-well plate. Colonies were counted after 7 days.

### Peptide fishing

Peptides corresponding to known or predicted tyrosine phosphorylation sites in FLT3 were imbobilized to the ultralink and used to pull-down GADS from COS1 cell lysates overexpressing GADS.

### Mouse xenograft model

Cells were washed three times with cold PBS to remove cytokins. NOD scid gamma (NSG) mice were injected with 200,000 cells with 1:1 matrigel subcutaneously. Tumor volume was measured twice a week and mice were sacrificed 23 days after injection.

### Microarray analysis

RNeasy mini kit (Qiagen) was used to extract total RNA from cells that were starved overnight. Affymetrix GeneChip Mouse Gene 2.0 ST arrays were used to analyze mRNA expression. Gene set enrichment analysis (GSEA) was used to analyze pathway enrichment.

### Confocal microscopy

Cells were stimulated at different time points before fixing using 2% paraformaldehyde in PBS. Cells were then blocked and permeabilized using a mixture of 0.5% Triton-X100 and 5% goat serum in PBS. Cells were stained using PE-conjugated anti-FLT3 and Alexa Flour 647-conjugated anti-FLAG antibody.
